# Supplementary material for: The Yin and Yang of Breast Cancer: Ion Channels as Determinants of Left–Right Functional Differences
Source: Int J Mol Sci. 2023 Jul 5;24(13):11121. doi: 10.3390/ijms241311121 (PMC10342022; doi:10.3390/ijms241311121)
Supplement: Supplementary file 1 [file ijms-24-11121-s001.zip › ijms-2420261- Supplementary Figures.pdf]

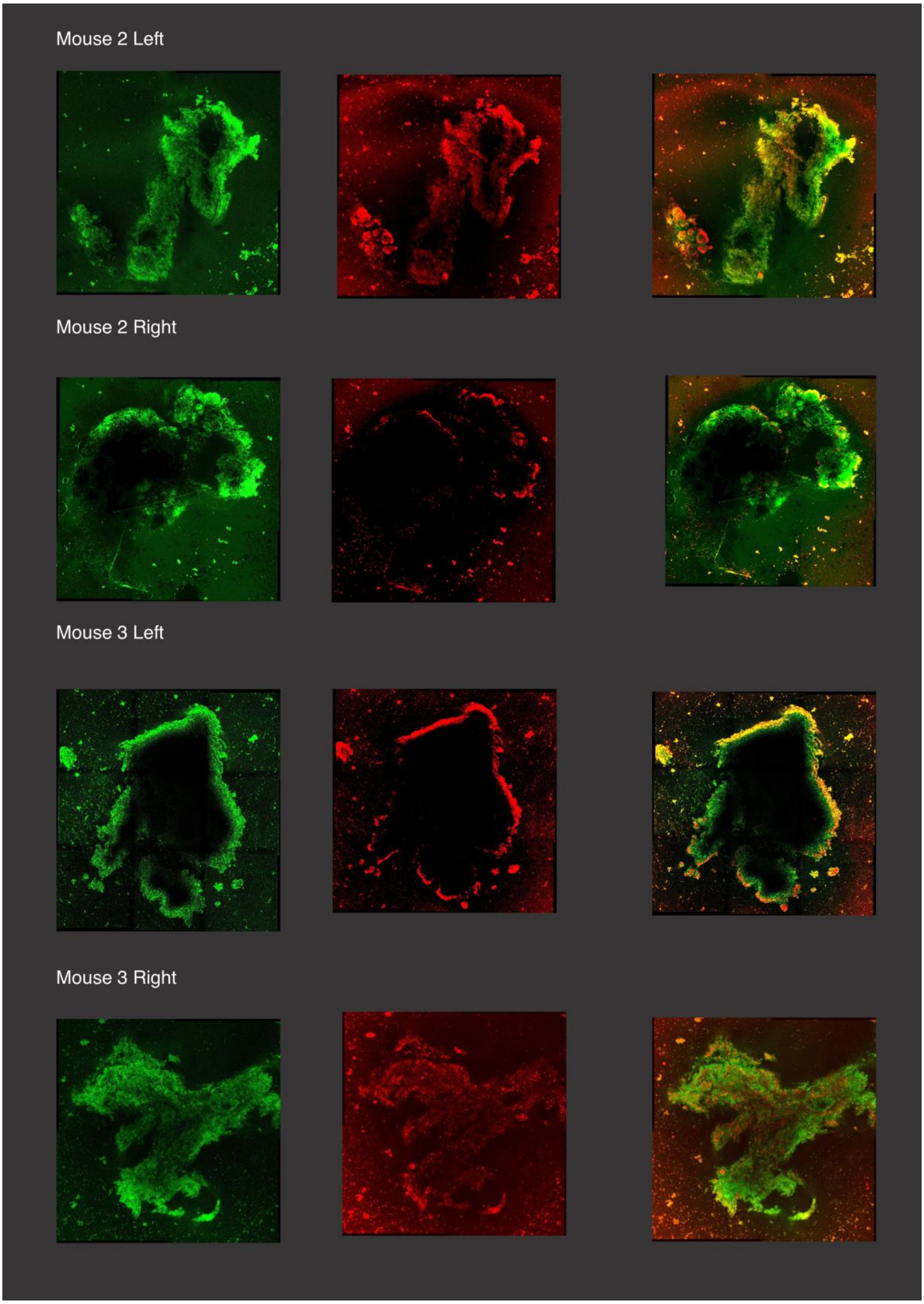

Supplementary Figure S1A

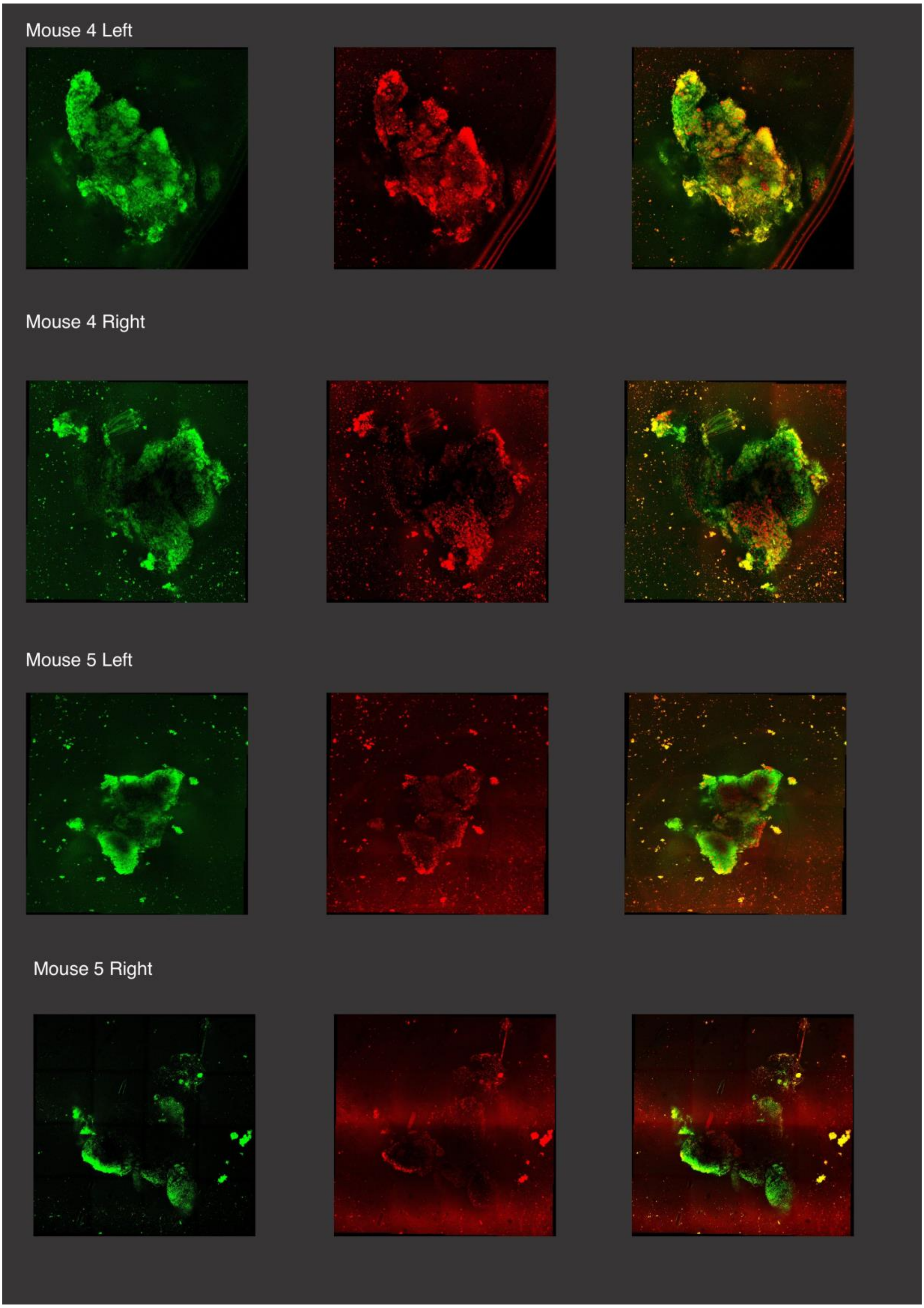

Supplementary Figure S1B
